# Advances in Liquid Metal-Enabled Flexible and Wearable Sensors

**DOI:** 10.3390/mi11020200

**Published:** 2020-02-15

**Authors:** Yi Ren, Xuyang Sun, Jing Liu

**Affiliations:** 1 Department of Biomedical Engineering, School of Medicine, Tsinghua University, Beijing 100084, China; ren-y18@mails.tsinghua.edu.cn; 2 Beijing Key Lab of CryoBiomedical Engineering and Key Lab of Cryogenics, Technical Institute of Physics and Chemistry, Chinese Academy of Sciences, Beijing 100190, China

**Keywords:** soft sensors, liquid metal, fabrication, principle, arrays, application

## Abstract

Sensors are core elements to directly obtain information from surrounding objects for further detecting, judging and controlling purposes. With the rapid development of soft electronics, flexible sensors have made considerable progress, and can better fit the objects to detect and, thus respond to changes more sensitively. Recently, as a newly emerging electronic ink, liquid metal is being increasingly investigated to realize various electronic elements, especially soft ones. Compared to conventional soft sensors, the introduction of liquid metal shows rather unique advantages. Due to excellent flexibility and conductivity, liquid-metal soft sensors present high enhancement in sensitivity and precision, thus producing many profound applications. So far, a series of flexible and wearable sensors based on liquid metal have been designed and tested. Their applications have also witnessed a growing exploration in biomedical areas, including health-monitoring, electronic skin, wearable devices and intelligent robots etc. This article presents a systematic review of the typical progress of liquid metal-enabled soft sensors, including material innovations, fabrication strategies, fundamental principles, representative application examples, and so on. The perspectives of liquid-metal soft sensors is finally interpreted to conclude the future challenges and opportunities.

## 1. Introduction

Sensors are common and core devices for information acquisition, which can detect external stimuli and transform them into measurable signals according to specific rules. In particular, biomedical sensors are targeted at human beings and other biological organisms to monitor physiological signals of life activity for health monitoring and disease diagnosis [1]. 

A typical sensor mainly consists of three components, including sensing element, conversion element and electrical circuits. The sensing element directly perceives the measured quantity and converts the signal into other values following a definite relationship, while the conversion element converts the non-electric quantity sensed by the sensitive element into electrical quantity [2]. Generally, the sensitive element and conversion unit are integrated as a whole. Diverse sensing materials are utilized to sense environmental change and produce a useful signal, such as piezoelectric effect, thermoelectric effect, Hall Effect etc. [2]. According to various detecting principles, existing sensors mainly include pressure sensors, force sensors, liquid sensors, speed sensors, acceleration sensors, radiation sensors, thermal sensors and so on [3,4,5,6,7,8,9].

With the rapid development of soft electronics [10,11,12,13,14,15,16,17,18,19,20], flexible and wearable sensors have shown evident advantages in biomedical applications, such as health-monitoring, wearable devices, artificial skin and intelligent robotics [10,21,22,23,24,25,26,27,28,29,30,31]. For instance, strain sensors can be attached to human body to monitor skin strain and muscle movement [32]. Introduction of soft sensors has greatly propelled the development of intelligent artificial skin, which provides not only aesthetic functions, but also perception of tactile sensation and temperature measurement [33,34]. They can also be applied to realize intelligent perception and control for robots [35,36,37]. Moreover, flexible sensors can tightly fit the object and maintain performance even while being stretched. In this way, they can respond to changes more sensitively and detect position and posture alteration in real time.

Up to now, materials and techniques to achieve flexible sensors have been widely explored. To obtain better application output for the human body, both flexibility and biocompatibility of the substrates are required. Soft thermoplastic polymers and silicone elastomers are the most common substrates used to fabricate flexible sensors, such as polyethylene terephthalate (PET), poly urethane (PU), polydimethylsiloxane (PDMS) and Eco Flex [38]. Moreover, the sensing elements are other important parts, which are mainly made up of conductive materials, including organic and inorganic nanomaterials [39,40]. Carbon-based materials, such as carbon nanotube and graphene, have been investigated in various soft circuits [39,41,42,43,44,45,46]. Although circuits fabricated by carbon-based materials are highly stretchable, they are not as conductive as those fabricated by metals. Nanoparticles of rigid metals are applied to design soft circuits with high conductivity, among which silver nanoparticles have been extensively studied [47,48,49,50,51,52,53]. Moreover, liquid metal, mainly refers to metal whose melting point is around the room temperature, is increasingly popular in the area of soft circuits because it possesses both good deformability and conductivity [54]. The room-temperature melting alloys can be fabricated by stirring in a liquid state, and sometimes heating is needed. For instance, the conductivity of gallium is $2.2\times 10^{6}\;S/m$, which is a little lower than that of silver but is much higher than that of organic materials [54]. Furthermore, liquid metal is more flexible and cheaper than silver. Thus, progress in liquid metal has been expected to propel the development of soft circuits. Typical liquid metal includes gallium, bismuth, lead, tin, cadmium, indium and their alloys. These low-melting alloys can be produced by stirring in a liquid state, and some raw materials with high melting points need to be heated. When exposed to the air, liquid metal will be oxidized immediately and form a layer of protective film. Unlike conventional liquid metal like mercury, gallium and its alloys is proven to be low-toxic and biocompatible, and has been introduced to biomedical area [55]. 

Due to the unique properties of liquid metal, it has been vigorously studied in soft electronics, such as flexible conductors, antennas, transistors, electrodes, energy-harvesting devices, sensors, liquid robots and liquid computational systems [56,57,58,59,60,61,62,63,64,65,66,67,68]. In particular, liquid-metal sensors are designed on the basis of variable resistance and capacitance during stretching and bending. Compared to nanoparticles of rigid metals, liquid metal appears more fluidic and deformable. Therefore, liquid-metal based sensors would fit well to the targeted area, further reducing discomfort and displaying better sensitivity and flexibility. The main problem that must be paid attention to liquid metal during application is the risk of leaking from soft encapsulation. 

In this review, the progress of soft sensors based on liquid metal is reviewed. First, we introduce the composition of liquid metal, as well as its distinct properties thus enabled. Next, fabrication methods of liquid-metal sensors and general comparisons among them are discussed. Subsequently, we move to interpret the basic principles of how liquid-metal sensors work. Following that, several typical sensors or sensor arrays based on liquid metal and their representative applications are illustrated. Finally, we point out the existing issues that would hinder further development in this area, and propose possible strategies for future research. 

## 2. Materials 

### 2.1. Composition

**Figure 1:**
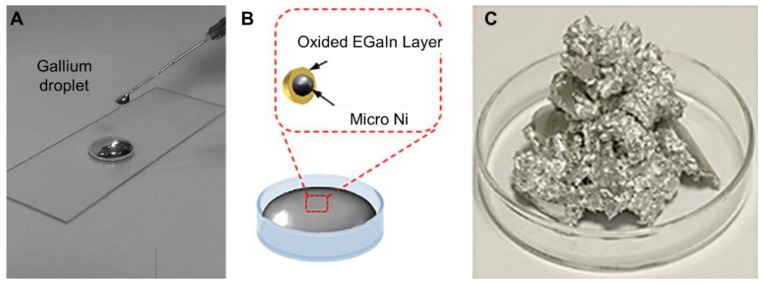
Liquid metal and its mixture. (**A**) Photo of gallium droplet, reproduced with permission from [69]. (**B**) Schematic diagram of Ni-GaIn particle, reproduced with permission from [70]. (**C**) Photo of Cu-GaIn mixure, reproduced with permission from [73].

At this stage, liquid metal applied to soft electronics mainly refers to gallium (Figure 1A) and its alloys, among which EGaIn (gallium and indium) and GaIinstan (gallium, indium and tin) are most commonly available [55]. EGaIn contains 75.5% gallium and 24.5 % indium by weight and its melting point is 15.5 °C, while GaIinstan consists of 68.5% gallium, 21.5% indium and 10.0% tin by weight and its melting point is 10.5 °C. Those alloys can be prepared by stirring in liquid state and sometimes heating is required. Similar to gallium, bismuth can be mixed up with other metals to form low-melting alloys as well. Their physical properties like melting point and electric conductivity can be regulated according to the addition of different metals [69].

In particular, the properties of liquid metal can be optimized by mixing liquid metal with other metal nanoparticles. The doping ratio decides the fluidity of the mixture. When the ratio of metal nanoparticles is high enough, the mixture will be in the form of a paste. For instance, magnetism, ferromagnetic nano powders could be dispersed in liquid metal [55]. Moreover, nickel particles could be mixed with EGaIn for fast fabrication of soft electronics by direct writing or printing [70,71,72]. As shown in Figure 1B, Ni particles were wrapped by a liquid metal oxide layer after stirring for a moment. The introduction of Ni particles could help enhance the adhesion force of liquid metal to substrate. Furthermore, to improve its conductivity, one could load copper particles into liquid metal to make ever superior composite [73]. Figure 1C shows the mixture of Cu-GaIn. The doping ratio and particle diameter could be adjusted according to different application requirements. Thermal and mechanical properties were tunable as well.

### 2.2. Property

**Figure 2:**
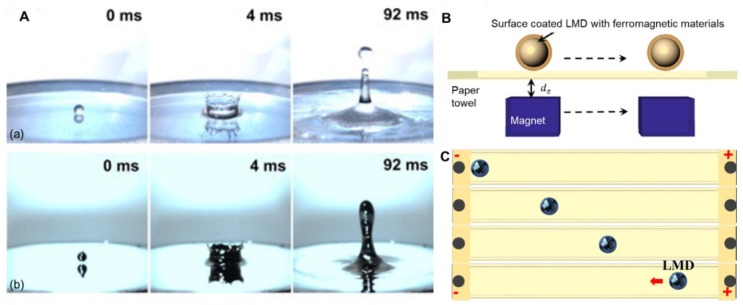
Typical properties of liquid metal. (**A**) Comparison of splashing process of water (**a**) and liquid metal (**b**), reproduced with permission from [74]; (**B**) Schematic of the magnet-controlled moving of an EGaIn droplet coated by ferromagnetic materials, reproduced with permission from [76]; (**C**) Sequential snapshots of a EGaIn droplet’s motion, reproduced with permission from [76];.

It is well known that liquid metal exhibits unique physical and chemical properties, which have been extensively studied. The most outstanding feature of liquid metal is that it has both fluidity and metallicity, making it advantageous to soft electronics. Previous experiments have testified that although liquid metal is fluidic, its dynamic performance is different from that of water, such as droplet shape and splashing morphology [74]. Figure 2A shows the splashing process of water and liquid metal under 25 °C. Clearly, it is hard for liquid metal to form secondary droplets due to high dynamic viscosity, which is really easy for splashing water. Moreover, gallium and its alloys could be oxidized quickly when exposed to air, which would lead to an even higher viscosity, increasing the difficulty to form secondary droplets. But on the other hand, formation of the oxide skin can increase the adhesive force, thus providing convenience for fabrication of liquid metal circuits. Research has found that liquid metal possesses a high surface tension and is hardly permeable on various substrate materials [75]. Metal particles can be mixed to regulate the adhesion and wettability of liquid metal, as mentioned above.

Liquid metal shows good electrical and thermal conductivity. The electrical conductivity of EGaIn is around $10^{6}\;S/m$ and the thermal conductivity is much higher than that of water. Moreover, when applying an electric or magnetic field, liquid metal would respond [76]. Coated with ferromagnetic materials, liquid-metal manipulation could be induced by an external magnetic field. As shown in Figure 2B, liquid-metal droplets could move following the magnet. An electrical field could induce the movement of liquid metal as well [77]. Figure 2C presents the movement of an EGaIn droplet under the control of an electrical filed. Furthermore, Tan et al. studied the movement of liquid-metal droplets with and without aluminum, respectively, and found that when there existed aluminum, liquid-metal droplets could run faster under the control of an electric field [78]. This is because that EGaIn and aluminum would constitute galvanic battery in electrolyte solution and the gas generated could propel the movement of the liquid metal. This redox can be represented by the chemical formula below:

(1) $$2Al+6H^{+}\;\to 2Al^{3+}+3H_{2}.$$

Since liquid metal shows these distinctive properties, the application of liquid metal in soft circuits is attracting increasing attention.

## 3. Fabrication

Owing to the unique physical properties of liquid metal, different methods to fabricate liquid-metal soft circuits have been designed and explored. Up to now, printing and microfluidic technologies are most commonly used [79]. Other methods including selective wetting, laser engraving, wiping etc., are investigated as well.

### 3.1. Printing Technology

**Figure 3:**
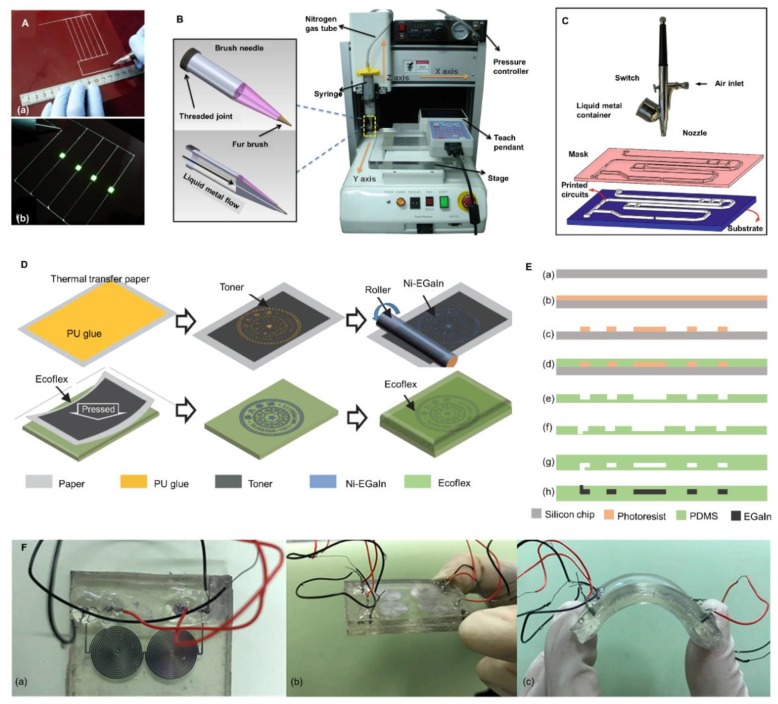
Printing and microfluidic technology. (**A**) Printing circuits with liquid metal ink by a roller ball pen, reproduced with permission from [82]. (**B**) Mechanical printing of liquid metal circuits with desktop printer, reproduced with permission from [84]. (**C**) Schematic of patterning liquid metal with a rigid mask, reproduced with permission from [90]. (**D**) Schematic of patterning liquid metal with toner mask, reproduced with permission from [72]. (**E**) Schematic of microfluidic technology: (**a**) The silicon chip; (**b**) Silicon chip coated by photoresist; (**c**) Lithography model; (**d**) Pouring PDMS; (**e**) PDMS with micro-grooves; (**f**) Punching; (**g**) Plasma bonding; (**h**) Injecting liquid metal. (**F**) A stretchable pressure sensor fabricated by the microfluidic technology: (**a**) Vertical view of the pressure sensor; (**b**) Original state of the pressure sensor; (**c**) A pressure sensor under bending; Reproduced with permission from [96].

Based on the fluidity of liquid metal, the concept of liquid-metal printed electronics was proposed [79,80]. Researches have proven that liquid metal can be used as ink and printed directly by a brush or a roller ball pen [81,82,83]. Compared to a traditional printed circuit board, this made electronics fabrication more convenient and rapid. Figure 3A shows how to paint a circuit with a roller ball pen and the light-emitting diodes (LEDs) are successfully lighted. To standardize the fabrication process of soft circuits by liquid metal ink and avoid artificial errors, automatic printing of liquid metal by a versatile desktop printer was further developed [84,85]. The concrete structure of a desktop printer with liquid metal ink is presented in Figure 3B. Circuits could be precisely designed by the desktop printer and then transferred to soft substrate, such as PDMS. Those circuits would be further encapsulated for practical application [86]. Votzke et al. designed a highly stretchable strain sensor using printed liquid metal, which has been used to measure elbow flexion angle [87]. Furthermore, Gannarapu et al. proposed the method of freeze-printing to fabricate 3D curvilinear paths. Liquid metal was continuously dispensed and frozen. The printed solid network could be encapsulated in elastomers [88]. A shortage of direct printing lies in that the resolution of circuits is sometimes limited by the resolution of the printer and can hardly be improved. To design liquid metal with smaller size, Liang et al. wrote liquid metal on a prestretched elastomeric substrate surface [89]. Then the prestretched substrate was released to recover the original size with electronic patterns shrinking on it.

Recently, mask-based printing has been designed as an alternative to promote resolution. For instance, a rigid mask could be fabricated by micromachining technology in advance. Areas covered by the mask would not be available to liquid metal and specific patterns could be deposited through the mask with an airbrush (Figure 3C) [90]. Moreover, a photoresist mask was commonly used as well, which can be removed finally, as well as the liquid metal falling on it [91,92]. However, unlike a rigid mask, a photoresist mask cannot be recycled and the process of photoetching is complex and time-consuming. Guo et al. introduced toner as the mask and liquid metal could selectively adhere to area without toner [72]. A roller was applied to deposit liquid metal and the circuit could further be transferred to elastic substrate. Figure 3D shows the whole process of Guo et al.’s method. 

In general, compared to other methods, printing technology may be more convenient, but the fabrication precision is relatively limited.

### 3.2. Microfluidic Technology

Meanwhile, microfluidic technology is advantageous for the fabrication of stable, uniform and sealed liquid-metal circuits [93,94,95,96,97,98,99]. Figure 3E is the schematic of the microfluidic technique. Firstly, photoetching was applied to produce a raised pattern model. Then, the mixture of PDMS prepolymer and curing agent with the quantity ratio of 10:1 was poured onto the model to form a PDMS layer with specific grooves. Plasma bonding was utilized to form sealed micro-channels. To fill the channels with liquid metal and exhaust air, a pair of inlet and outlet holes must be produced before plasma bonding. After that, liquid metal could be injected into the channels. Circuits fabricated by microfluidic technology showed excellent stability and uniformity. Microfluidic technology has already been applied widely and commercially.

Several types of soft force sensors have, thus, been designed by this method. As shown in Figure 3F, the soft pressure sensor was fabricated by microfluidic technology. Research has proven that the soft force sensors could generate a response to external pressure through the change of capacitance and exhibit good flexibility.

### 3.3. Selective Wetting

Not limited to printing and microfluidic technology, fabrication methods of flexible sensors based on liquid metal are diverse due to the unique properties of liquid metal [79]. For instance, considering that the wetting behavior of liquid metal varies with the surface properties of the substrate, selective wetting of liquid metal is studied. 

**Figure 4:**
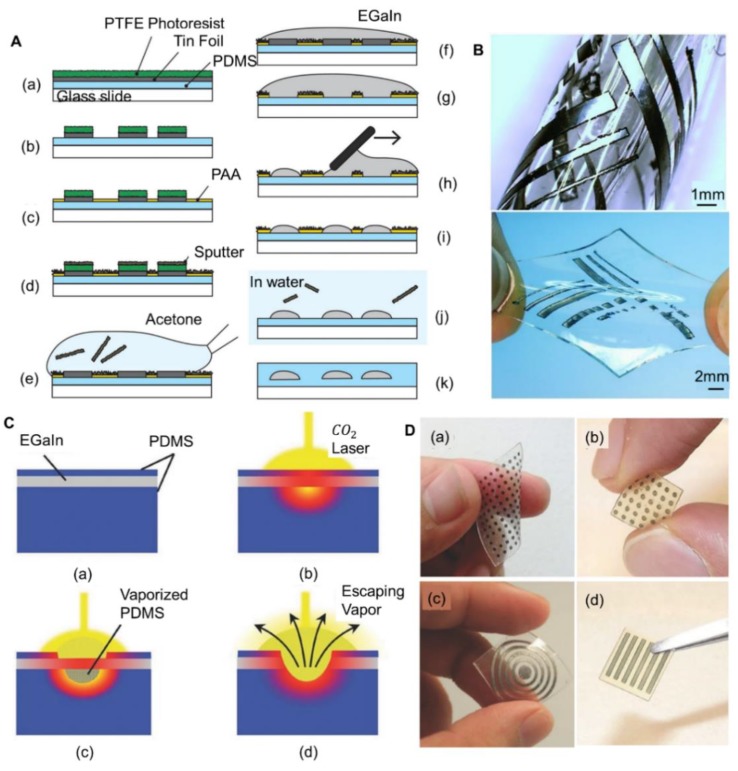
Diverse techniques to fabricate liquid-metal soft electronics. (**A**) Fabrication process of selective liquid metal deposition: (**a**) Photoetching; (**b**) Photoresist developing; (**c**) Spinning poly acrylic acid (PAA); (**d**) Sputtering the surface with indium; (**e**) Removing photoresist; (**f**,**g**) Flooding the surface with liquid metal; (**h**,**i**) Removing excess liquid metal; (**j**) Removing indium mask; (**k**) Encapsulating the circuit. Reproduced with permission from [100]. (**B**) A circuit embedded in elastomer patterned by selective deposition of liquid metal, reproduced with permission from [100]. (**C**) Illustration of CO_2_ laser engraving technology, reproduced with permission from [102]. (**D**) Flexible circuits fabricated by CO_2_ laser, reproduced with permission from [102].

Kramer et al. had fabricated elastomer conductors by deposition, which was based on the different wetting behavior between liquid metal and thin metal films [100]. Figure 4A shows the whole process of fabricating circuits by this method. The surface was sputtered with indium, which prevented liquid metal from adhering to this area. As shown in Figure 4B, the circuits thus fabricated on a soft substrate were proven to be highly flexible. Similarly, Li et al. presented a stretchable pulse sensor fabricated by selective plating as well, in which a thin film of copper would be electroplated in advance to enhance the wetting of liquid metal [101]. Clearly, surface modification could be utilized by either enhancing or weakening the wettability of liquid metal so as to form designed patterns. The limitation of this method may be that the process of surface modification is complex and the edge of the pattern produced by selective wetting is rough.

### 3.4. Laser Engraving

The laser engraving technique is commonly applied in the field of micromachining. CO_2_ laser engraving technology was introduced for rapid prototyping of soft electronics as well [102]. Figure 4C shows the direct patterning of liquid metal by a CO_2_ laser cutter. Firstly, a thin layer of liquid metal was sealed by two layers of PDMS and the top layer mainly functioned as protection during the laser-cutting process. PDMS could absorb the energy from the CO_2_ laser and be vaporized. Then liquid metal would escape together with PDMS vapor, thus forming desired patterns. After that, a new layer of PDMS would be cast to encase the circuit. Lu et al. have designed several systems through this technology, including sensors and resistive circuits (Figure 4D). CO_2_ laser-engraving technology is advantageous for the fabrication of any 2D structure. However, it causes a higher wastage of liquid metal since a large proportion of liquid metal escaped with vaporized PDMS, which could be avoided by collecting the liquid metal that escaped. Recently, NdYAG lasers were applied for simpler patterning of liquid metal [103]. Pan et al. produced a film composed of a liquid metal grid through direct laser writing, and proved that the circuit showed high optical transmittance, high stretchability, low resistivity and low trace width.

### 3.5. Dewetting and Wiping

**Figure 5:**
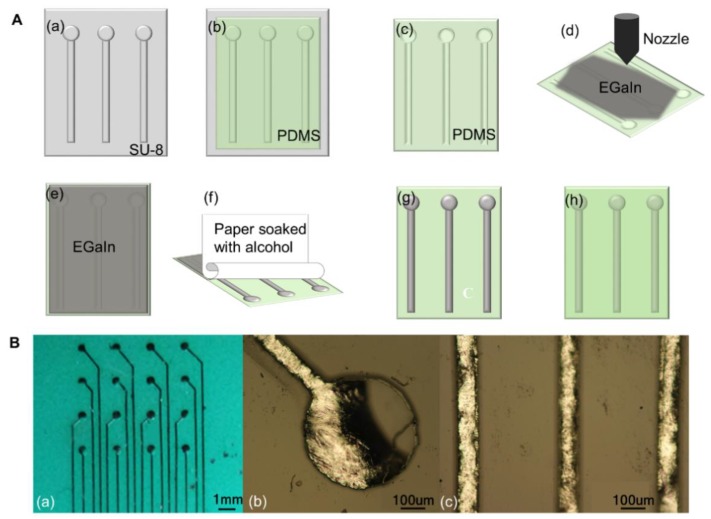
Technologies of spraying and wiping. (**A**) Fabrication process: (**a**) Photolithography model; (**b**) Pouring PDMS onto the photolithography model; (**c**) PDMS substrate with micro-channels; (**d**,**e**) Spraying liquid metal with an airbrush; (**f**) Removing needless liquid metal with the help of paper soaked with absolute ethyl alcohol; (**g**) The final circuit; (**h**) Encapsulating the circuit. (**B**) An electrode array fabricated by spraying and wiping: (**a**) The electrode array; (**b**) The micrograph of the electrode plate; (**c**) The micrograph of lines. Reproduced with permission from [105].

Meanwhile, methods of dewetting [104] and wiping [105] utilized the property that liquid metal would be oxidized immediately and form a thin oxide layer when exposed to the air or oxygen. The oxide layer could increase the adhesion between liquid metal and micro-channels. Therefore, while liquid metal outside the channels was removed, liquid metal in the channels would be left owing to the higher adhesion force. The difference is that dewetting uses NaOH solution to remove excess liquid metal and wiping uses absolute ethyl alcohol (Figure 5A). Compared to dewetting by NaOH solution, wiping by absolute ethyl alcohol is more controllable since the reaction between NaOH solution and liquid metal is more intense.

As shown in Figure 5A, the first few steps of wiping were similar to microfluidic technology: photoetching was used to form the desired pattern model and micro-grooves. But inlet and outlet holes were unnecessary, which significantly simplified the fabrication process and improved the resolution. After spraying liquid metal onto PDMS by a nozzle, paper soaked with absolute alcohol was applied to remove excess liquid metal. The circuits could be encapsulated by casting another layer of PDMS. Figure 5B (a) is an electrode array with high resolution fabricated by spraying and wiping. Figure 5B (b),(c) show the detailed micrograph of the electrode and lines. It could be concluded that the channels were fully filled by liquid metal. A higher failure rate may be the main limitation of the wiping technique, but this can be made up by increasing the depth of micro-channels and repeating the operations of spraying and wiping.

## 4. Basic Principle of Liquid-Metal Sensors

### 4.1. Liquid Metal as Soft Connection

**Figure 6:**
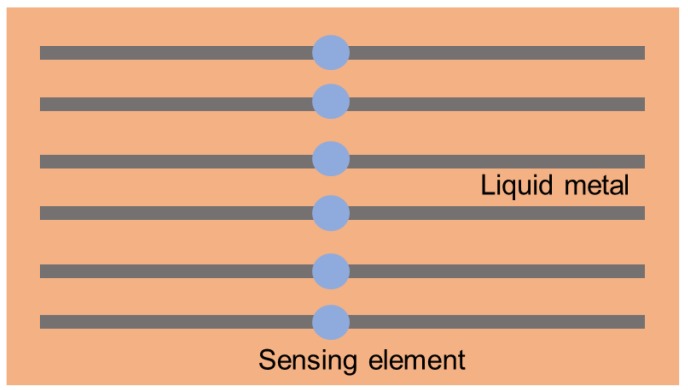
Illustration of circuits using liquid metal as soft connection.

The simplest liquid-metal sensors are perhaps those that use liquid metal as soft connections (Figure 6). The detecting mechanism is the same as rigid ones, but the stretchability is greatly improved. Liquid metal possesses both good conductivity and fluidity, ensuring the reliability of the connections during stretching and bending. For instance, based on photoplethysmography (PPG) technology, Li et al. designed a stretchable pulse biosensor with liquid-metal interconnections and soft substrate [101]. This system was proven to be convenient and comfortable for heart-rate monitoring. In addition, Hong et al. designed a soft polyaniline nanofiber temperature sensor array, whose interconnections were made up of Galinstan [106]. It could be used as a portable and wearable device to measure body temperature. Combining liquid-metal wiring with graphene sensors, Jiao et al. developed several strain sensors, which could be used for health-monitoring and motion-sensing [107]. Meanwhile, Hu et al. put forward the idea of using liquid metal to connect the sensing element and successfully produced a sensor array, which had the ability to sense temperature and force [108]. All these research works prove that a liquid-metal connection provides advantages in fabricating flexible sensors.

### 4.2. Resistive Sensors

**Figure 7:**
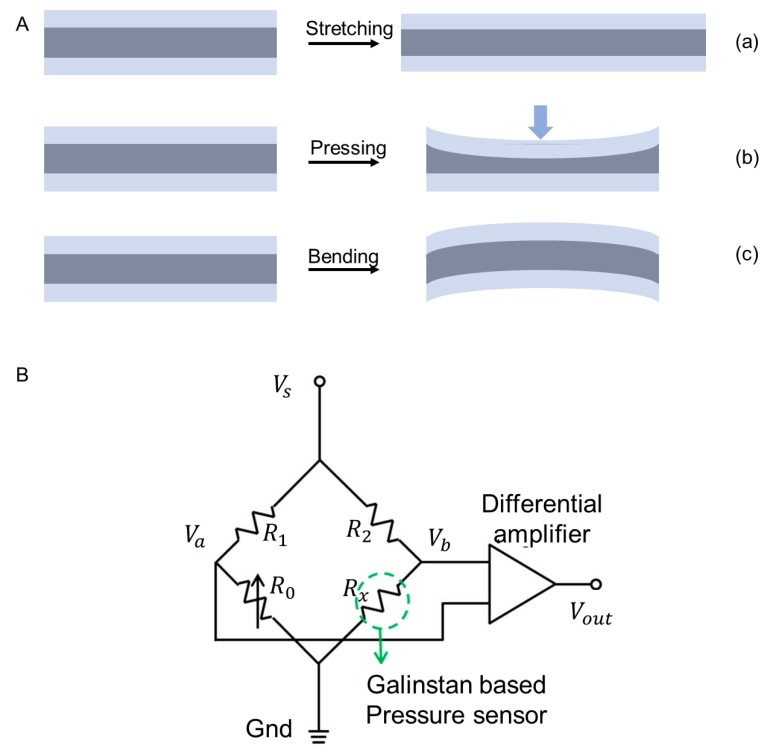
Working principle of resistive sensor. (**A**) Deformation of liquid metal under external force. Blue represents soft substrate and gray represents liquid metal: (**a**) Stretching; (**b**) Pressing; (**c**) Bending. (**B**) Wheatstone bridge to detect the resistance of the liquid-metal pressure sensor, reproduced with permission from [95].

Resistive sensors based on liquid metal have been widely researched. External change can be reflected through the fluctuations in resistance. The resistance *R* follows the formula:

*R* = *ρL*/(*S*), (2)

where *ρ* is the resistivity of liquid metal; *L* is the length and *S* is the cross-sectional area. External heat and force can lead to the deformation of liquid metal, including thermal expansion, stretching and pressing, which further causes a change in resistance. As shown in Figure 7A, stretching can influence the length and cross-sectional area of liquid-metal lines, while pressing can change the cross-sectional area. When being bent, the shape of liquid metal does not change significantly, as well as the resistance.

Generally, liquid-metal resistive sensors include temperature sensors and force sensors. For instance, Li et al. has found the matching metal of liquid metal and designed a printable tiny thermocouple base on them. This thermocouple showed an excellent linear property when the temperature ranged from 0 to 200 °C [109]. Likewise, some force sensors were fabricated on the basis of resistive response, including pressure sensors [95,110], strain sensors [97,111] and shear force sensors [111]. To accurately measure the change of resistance, several specific circuit organizations were applied. Figure 7B is the schematic of a Wheatstone bridge with a Galinstan based sensor. The application of a Wheatstone bridge helps detect resistance of the sensor with high precision. When the voltage $V_{a}$ is equal to $V_{b}$, which means that the output sensor $V_{out}$ is zero, the resistance of the Galinstan based sensor can be calculated by the following equation:

(3) $$R_{x}=\frac{R_{2}}{R1}R_{0}$$

$V_{out}$ is the amplification of $V_{b}-V_{a}$. This circuit can sensitively reflect the change of voltage and thus the resistance of the sensor. For instance, when applying external force to the Galinstan-based pressure sensor, deformation of the soft circuit would cause the change of resistance, which further influences the output voltage $V_{out}$. The resistance of $R_{0}$ should be adjusted until $V_{out}$ is zero again. Then the new resistance of the pressure sensor can be calculated, as well as the external force. 

### 4.3. Capacitive Sensors

**Figure 8:**
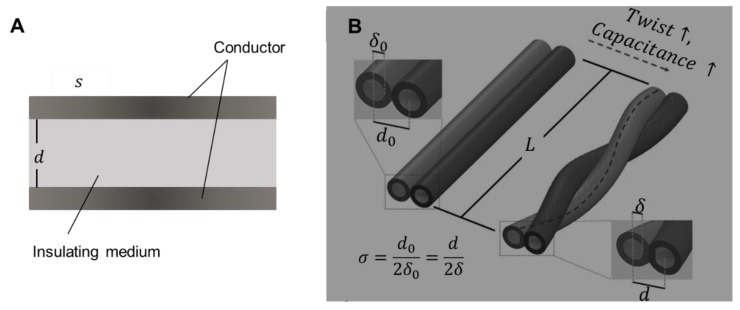
Working principle of capacitive sensor. (**A**) Illustration of capacitor: s is the facing area and d is the vertical distance. (**B**) Stretchable capacitive sensors with double helix liquid metal fibers, reproduced with permission from [114].

Some capacitive force sensors have been designed as well, which mainly work based on the variation of capacitance [66,112,113,114,115]. The theory of capacitive sensors is similar to that of resistive sensors. In principle, the capacitance is decided by the formula below:

*C* = (ε*S*)/*d*, (4)

where ε is the dielectric constant; *S* is the facing area and *d* is the vertical distance (Figure 8A). When applying an external force, parameters of the capacitor will be altered, causing the change of capacitance. 

More complex and precise sensors can be fabricated through the series and parallel combination of capacitors. Figure 8B is a stretchable sensor with two liquid metal fibers, whose capacitance would change when being twisted. It could be used to measure torsion, strain and touch through the change of capacitance. Moreover, an inertial sensor based on a liquid metal droplet was designed as well. The liquid droplet was not fixed, so that it could modulate the capacitance between electrode 1 and 2 during movement. Compared to rigid sensors, these capacitive sensors based on liquid metal has good flexibility and stability.

### 4.4. Electrochemical Sensors

Electrochemical sensors utilize the chemical reaction occurring in solution. EGaIn shows good electrochemical behavior. Liquid metal with nickel and aluminum has been proven to be propelled by hydrogen in NaOH solution [78,116]. The movement can be controlled by an external electrical or magnetic field. Combined with saline solution, liquid metal can oxidize quickly and become gray gradually. Based on these reactions, liquid metal can be used to detect the existence of some substance and electrical field.

Moreover, Sivan et al. found that liquid-metal marbles could enhance the sensitivity of heavy metal ion sensors [117]. They produced liquid-metal marbles with controlled coating density and proved that these marbles showed enhanced electrochemical sensitivity towards heavy metal ions with excellent selectivity. It had been used to detect some specific heavy metal ions, such as $Pb^{2+}$ or $Cd^{2+}$. Compared to resistive sensors and capacitive sensors, studies on electrochemical sensors are relatively fewer.

### 4.5. Metamaterial Biosensors

**Figure 9:**
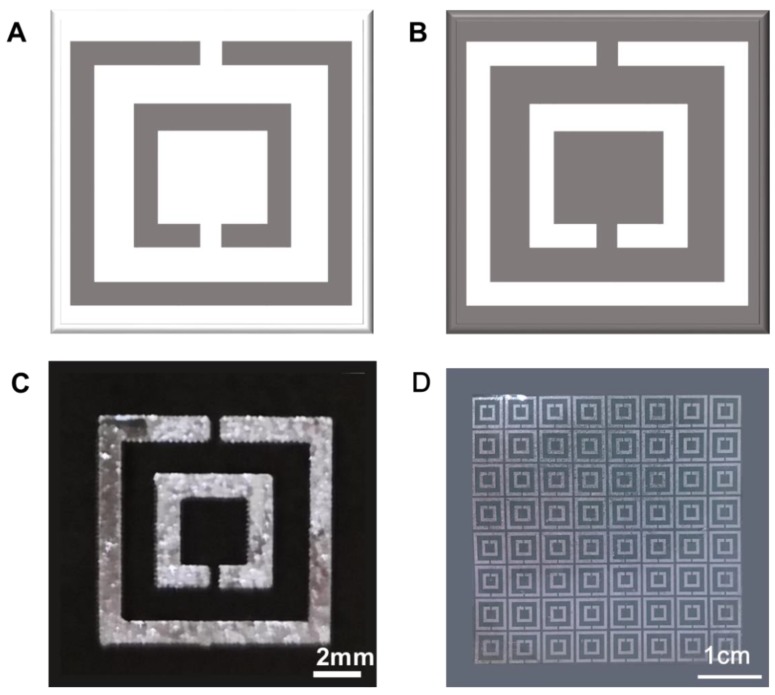
Metamaterial. Schematic diagram of split-ring resonator (SRR) (**A**) and complementary split-ring resonator (CSRR) (**B**), where the gray color represents metal and the white represents gaps. (**C**) An SRR unit fabricated by liquid metal. (**D**) SRR arrays fabricated by liquid metal.

With the development of metamaterials, liquid-metal metamaterials are on the rise. Among all the metamaterials, split-ring resonator (SRR) arrays and complementary split-ring resonator arrays (CSRR) are the most commonly used structures for electromagnetic metamaterials, as shown in Figure 9A,B. Single SRR unit and SRR arrays based on liquid-metal have been fabricated successfully by printing (Figure 9C,D). All those results prove that liquid-metal based metamaterials are possible. The primary limitation of a liquid-metal SRR structure is that the fabrication size is not fine enough to generate electromagnetic response. Besides SRR and CSRR structures, several other types of liquid metal electromagnetic metamaterials have been proposed as well. For instance, Xu et al. fabricated a switchable metamaterial exploiting liquid metal [118]. The interference between copper and liquid metal resulted in an electromagnetically induced transparency (EIT)-like spectrum, which could be switched on or off on the basis of the fluidity of liquid metal. Moreover, a flexible metamaterial absorber based on liquid metal and PDMS was proposed [119,120,121]. Reichel et al. also designed a terahertz signal-processing device with liquid metal, which was electrically reconfigurable [122].

Recent studies have also applied metamaterials to biosensors for molecule detection. The sensing mechanism of metamaterials is that a change in the resonant frequency would happen when different biomolecules bind onto metamaterial resonators [123]. It is hoped that metamaterials can be combined with with liquid metal to design soft metamaterial biosensors. Generally, research on electromagnetic metamaterials based on liquid metal are still in the initial stage and more efforts are needed. Upcoming research could be conducted to find out the feasibility of liquid-metal metamaterial biosensors.

### 4.6. Liquid-Metal Antenna

Antennas are important components in wireless devices for remote communication and sensing [124]. Several flexible antennas fabricated by liquid metal have been designed for implanted medical devices, interactive gaming and aeronautic remote sensing to avoid the mechanical failure of solid parts due to material fatigue, creep or wear [124,125,126,127]. A radiation pattern reconfigurable antenna was designed by Rodrigo et al., which could operate at 1800 MHz with 4.0% bandwidth [124]. Mazlouman et al. produced a frequency-reconfigurable antenna as well by injecting liquid metal into a square reservoir. Their antenna allowed frequency tuning from 1.3 to 3 GHz. Hayes et al. proposed a flexible antenna with a novel multi-layer construction based on liquid metal [126]. It was proven to be mechanically flexible and perform stably during flexing. Cheng et al. fabricated a flexible planar inverted cone antenna for ultrawideband applications [127]. It is expected that antennas based on liquid metal with improved durability and stability will be presented in the near future.

## 5. Typical Applications

Sensors play an important role in biomedicine and bionic area. So far, the application of soft sensors includes force sensors, temperature sensors, blood glucose sensors and so on. Based on the fluidity and metallicity of liquid metal, sensors enabled from them possess good flexibility and sensitivity. Obviously, sensors fabricated by liquid metal will become critical components for wearable devices and robots.

### 5.1. Force Sensors

Soft force sensors based on liquid metal, which can tolerate large deformation, have shown advantages in force detection and motion-monitoring. 

**Figure 10:**
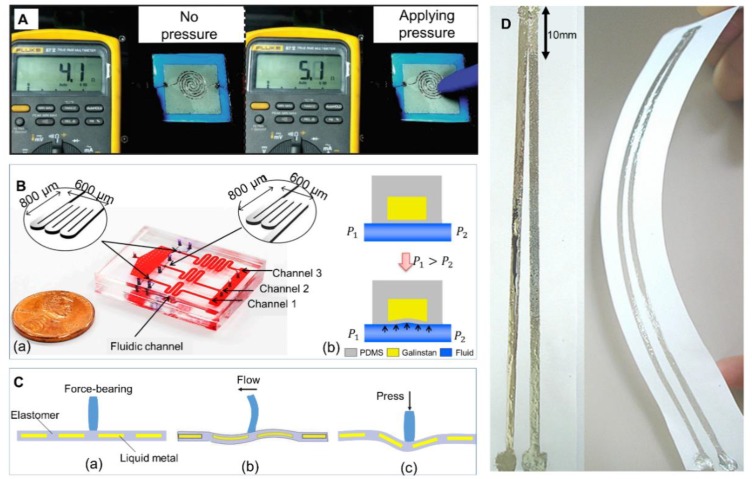
Photos of force sensors, temperature sensors and blood glucose sensors. (**A**) The resistance of a printed pressure sensor according to external pressure, reproduced with permission from [110]. (**B**) A micro-channel pressure sensor: (**a**) Structure of the sensor; (**b**) Working principle of measuring viscosity of various fluids; Reproduced with permission from [95]. (**C**) Schematic of artificial hair cell sensor: (**a**) No external force; (**b**) Deformation under a shear force; (**c**) Deformation under a normal force. (**D**) Thermocouple based on liquid metal printed on paper, reproduced with permission from [109].

For instance, pressure sensors have been researched vigorously. Previous experiments showed that when directly applying pressure to a printed liquid metal pressure sensor, the resistance would change accordingly [110]. As shown in Figure 10A, when there existed external pressure, the resistance of the liquid-metal sensor would increase. Moreover, Jung et al. manufactured a pressure sensor embedded in a microfluidic channel, which could be used to measure the viscosity of various fluids [95]. The change of the fluids’ viscosity would lead to a pressure to the membrane and then the electrical resistance would increase due to the decrease in the cross-sectional area (Figure 10B). A Wheatstone bridge circuit was applied in their research to detect the change of electrical resistance accurately. Kim et al. integrated liquid metal microchannels with a 3D-printed microbump array to increase the sensitivity of the pressure sensor, which has been proven to have a wide range of applications in health monitoring [93]. Oh et al. injected liquid metal into PDMS and designed a pressure-conductive rubber sensor. This could be used to detect wrist pulse and respiration [128]. Besides pressure sensors, strain sensors are also important types of force sensors. For instance, Zhou et al. fabricated a flexible strain sensor with asymmetric structure. This could detect various human joint motions simultaneously [129].

Vogt et al. have designed a multi-axis force sensor with microfluidic channels recently [106]. This could detect not only normal shear force, but also in-plane shear force. Meanwhile, Shi et al. reported a flexible force sensor to detect normal and shear forces as well based on the piezoresistive effect and further designed an artificial hair cell sensor [130]. Figure 10C shows the working principle of the hair cell sensor. The polymer hair would be deformed when there was a shear or normal force, causing the deformation of circuits connected to the hair. The change of capacitance between liquid metal could reflect the movement of polymer hair. It perfectly imitated the operating mode of hair cells in the inner ear, which might further propel the research of an artificial ear.

Cooper et al. proposed a stretchable capacitive sensor with double-helix liquid-metal fibers, which was capable of detecting torsion, strain and touch [114]. When being twisted or elongated, the geometry of fibers as well as the capacitance between the fibers would change accordingly. Moreover, a soft inertial sensor was introduced by Varga et al. based on the alteration of capacitance between two electrodes when the liquid-metal droplet moved [66]. This inertial sensor was sensitive to motion and position detection.

Recently, wearable transient epidermal sensors were fabricated by liquid-metal hydrogel [131]. Liquid-metal marbles were distributed evenly into polyvinyl alcohol by sonication to form liquid metal hydrogel. This material was flexible and self-healing. Epidermal sensors made by it could detect some human activities, like swallowing and finger bending.

Furthermore, soft force sensors are expected to be applied to electronic skin and intelligent robots to further promote the function of artificial products. Combining the advantages of traditional artificial skin and sensors, a soft electronic skin can provide not only aesthetic function, but also a perception function [132,133].

### 5.2. Temperature Sensors 

Several types of temperature sensors based on liquid metal have been studied. Hong et al. applied liquid metal as the interconnection between temperature-sensing units to fabricate soft temperature sensor arrays [106]. Their sensor array could be easily attached to the skin, which was important for electronic skin as well. A thermocouple based on liquid metal was proposed by Li et al. [109]. Ga with 0.25 wt. % Ga oxides and EGaIn21.5 with 0.25 wt. % Ga oxides were chosen as the thermocouple elements. The thermocouple was directly printed on paper (Figure 10D) and could be bent and twisted to some extent. 

Currently, temperature sensors based on liquid metal have not been investigated deeply because compared to existing rigid metal temperature sensors, liquid metal temperature sensors are not sensitive and convenient enough. Tremendous efforts will be made in the near future.

### 5.3. Blood Glucose Sensors

Yi et al. have designed an electrochemical sensor for wireless glucose-detecting based on liquid metal [134]. They fabricated liquid-metal electrodes by direct hand printing on polyvinyl chloride (PVC) substrate. In their research, those electrodes were modified by enzyme to detect glucose. When the enzyme reacted with glucose, the electrodes could transfer it to an electrical signal. Overall, this flexible sensor has been proven to be reliable by cycle voltammetry. They also designed a wireless system to transmit testing data to a smart phone via Bluetooth.

### 5.4. Sensor Array

An array is a common circuit arrangement for sensors, which can improve their sensitivity and accuracy. For instance, the temperature sensors [106] and the pressure sensor [95] mentioned above are all in the form of arrays. Specially, Zhang et al. developed a sensor array for application in robotic electronic skin [135]. They used cross-grid liquid-metal layers as electrodes. Both capacitance mode and triboelectric nanogenerators (TENG) [136] mode could be realized by this sensor array. There were two layers of cross-grid arrays constructed by liquid-metal electrodes. The capacitance between the top and bottom electrodes would increase or decrease when the environment changed. The adoption of liquid metal made the whole device highly flexible. It could function as an E-skin sensor with complementary capacitance mode or TENG mode, respectively. Compared to a single sensing element, sensor arrays can provide large-area and multi-functional detecting, especially for human health-monitoring.

### 5.5. Pneumatic Artificial Muscles

**Figure 11:**
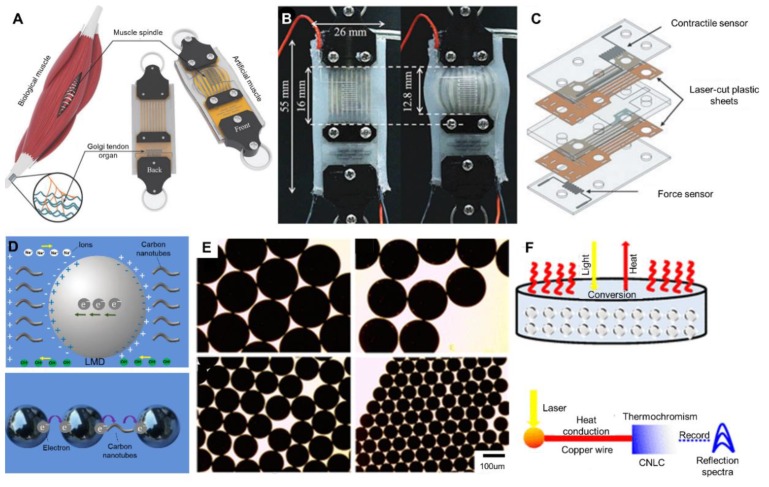
Artificial muscles and liquid metal microsphere sensors. (**A**) Schematic comparison between biological muscle and artificial muscle, reproduced with permission from [136]. (**B**) The sensorized, flat, pneumatic artificial muscle at rest and inflated to 82.8 kPa, reproduced with permission from [137]. (**C**) Illustration of the contraction and pressure sensors, reproduced with permission from [137]. (**D**) The electrical environment of a liquid-metal microsphere in NaOH solution (above) and two methods of communicating for liquid-metal microspheres in a circuit (below), reproduced with permission from [77]. (**E**) Microscopy images showing liquid-metal microspheres produced by capillary-based microfluidics, reproduced with permission from [77]. (**F**) Schematic diagram of the photothermal conversion of liquid-metal microspheres and the near-infrared (NIR) sensor based on the liquid metal microsphere, reproduced with permission from [138].

Pneumatic artificial muscles have been increasingly investigated owing to the potential in wearable devices. Inspired by biological muscle, Jackson et al. designed an artificial muscle with both force and position sensors [137], as shown in Figure 11A. The Golgi tendon organ and muscle spindle were fabricated by EGaIn. Figure 11B shows the form alterations when the sensorized, flat, pneumatic artificial muscle was at rest and inflated to the pressure of 82.8 kPa. Liquid metal was injected into micro-channels to form contractile and force sensors (Figure 11C). It has been proved that this artificial muscle could mimic the behavior of biological muscle well. The progress in artificial muscle is meaningful to wearable devices, as well as soft robots.

### 5.6. Liquid-Metal Microsphere Sensors 

The potential application of liquid-metal microspheres is increasingly attractive as well. The concept of liquid-metal enabled droplet circuits in solution was proposed previously [77]. Liquid metal droplets, ions and carbon nanotubes were combined in electrolyte solution to enhance electrical conductivity. Figure 11D shows the electric charge distribution of a liquid-metal microsphere in NaOH solution, and the communication methods between liquid-metal microspheres and carbon nanotubes in a circuit. The quantum tunneling effect is the fundamental principle lying behind liquid-metal droplet circuits. Besides the theoretical liquid-metal droplet circuits, sensors based on liquid-metal microsphere were researched recently [138]. Wei et al. designed a method to fabricate liquid-metal microspheres with different sizes through a capillary effect, as shown in Figure 11E. The size of liquid-metal microspheres could be precisely and stably controlled by the co-flowing configuration in the micro-channels. These liquid-metal microspheres had been proven to exhibit outstanding photothermal properties, which could be utilized to produce optical sensors, such as near infrared ray (NIR) sensors (Figure 11F). There is a photo-thermal conversion in liquid metal microsphere, which is promising to not only liquid-metal optical sensors, but also light-driven actuators and as an energy conversion medium. Moreover, Zhang et al. presented a flexible capacitive sensor based on liquid-metal microsphere as well [138]. It worked through the multi-plateau capacitance waveform when liquid metal sphere passed through the sensing area. It is believed that more applications of liquid-metal microspheres could be explored.

## 6. Perspective

In the near future, we may expect the appearance of metal-enabled liquid sensors. By contrast with traditional rigid and soft sensors, metal-enabled liquid sensors will be in the form of fluidity on the basis of liquid metal. The environment of the human body is liquid as well and all organs work in this environment. According to the ideas of bionics, research on liquid sensors may be an inevitable trend.

### 6.1. Concept of Liquid Sensors

**Figure 12:**
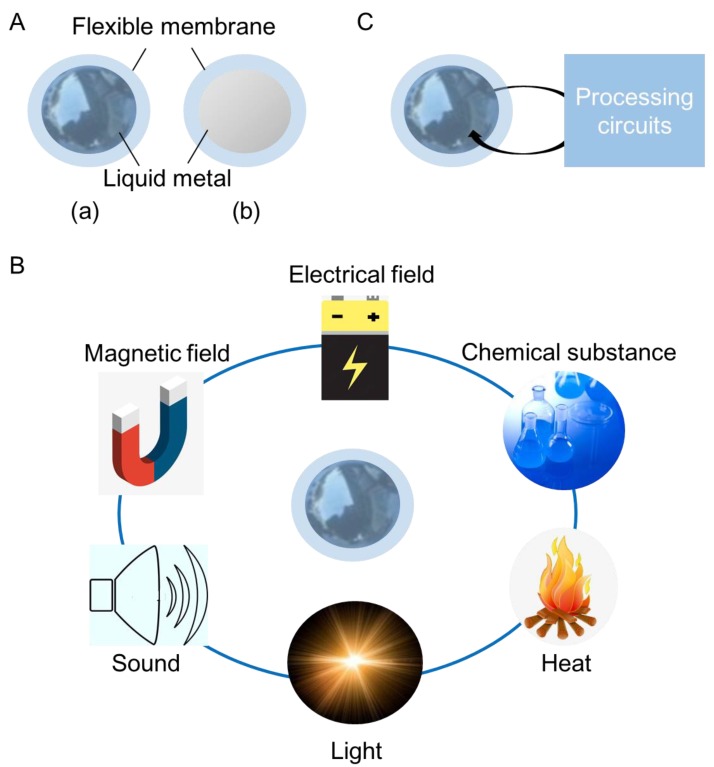
Metal-enabled liquid sensors. (**A**) Illustration of liquid sensor: (**a**) Front view; (**b**) Sectional view. (**B**) Working principle of liquid sensor. (**C**) Diagram of a liquid sensor system.

As shown in Figure 12A, a liquid sensor is fabricated by wrapping liquid metal with a flexible membrane. Liquid metal inside the membrane has greater freedom than that printed on soft substrate or injected into micro-channels. Thus, the liquid sensors are more flexible and transformable. To ensure good mechanical property of liquid sensors and avoid leakage of liquid metal, the membrane must exhibit excellent flexibility and compactness. Figure 12B gives the details of a liquid-sensor system. Processing circuits are required in order to amplify, transform and filtrate the electrical signal output by the liquid sensors. Then, the processed signal could be measured by the detecting device. Compared to existing soft sensors, liquid sensors are more suitable for in vivo detection because it is highly transformable. Injectable electrodes based on liquid metal have been proposed for tumor treatment already [65]. It is possible to design injectable liquid sensors with the application of liquid metal.

### 6.2. Working Principle of Liquid Sensors

Metal-enabled liquid sensors can make a response to external force, electrical field, magnetic field, sound, light, heat and some chemical substances, on the basis of the unique physical and chemical properties of liquid metal (Figure 12C). External stimulations will result in the changes of resistance or capacitance, which can be converted to measurable electrical signals, such as voltage or current signal. To reflect the alteration of the environment, the liquid sensors should be connected to a processing unit, as shown in Figure 12C. 

Qualitative liquid sensors based on liquid metal are not hard to realize. The difficulty is how to create a quantitative liquid sensor with high sensitivity and precision. More efforts are required to propel the development of liquid sensors.

## 7. Discussion 

Liquid metal is characterized by good fluidity, perfect metallicity and unique electromagnetic properties. Liquid metal-based soft sensors have attracted increasing attention. Generally, soft sensors fabricated by liquid metal imply the following advantages.

Firstly, sensors fabricated by liquid metal own excellent flexibility owing to the fluidity of liquid metal. They can remain working when being twisted, stretched and bent within limits. The existence of liquid metal ensures that the circuits are still connected during deformation and improves the reliability of those soft sensors. This allows the application of liquid-metal sensors in wearable devices with great promise.

Secondly, fabrication methods of liquid-metal based sensors are diverse. Due to the unique physical and chemical properties of liquid metal, patterning methods includes printing, microfluidic technology and others. Compared to traditional micro-/nano-machining technology, these new methods are convenient and economical. However, the resolution should be further improved by optimizing the fabrication process.

Last but not least, liquid metal-based sensors provide better conformability and sensing sensitivity. Since the flexibility of liquid metal circuit is guaranteed, it can well fit different parts of human body and promote the wearing experience. Furthermore, since sensors based on liquid metal can be tightly attached to objects to be tested, the sensing sensitivity is higher than that of rigid sensors or common soft sensors.

So far, liquid metal-based sensors have been vigorously researched because of their outstanding performance. It is hoped that more soft sensors based on liquid metal, especially metamaterial biosensors, can be designed. Through increasing endeavors, more sensing mechanisms enabled from liquid metals are also possible in the near future. Microsphere sensors based on liquid metal, and even metal-enabled liquid sensors, may cause a revolution in the development of soft sensors and in vivo human signal monitoring.

## 8. Conclusion

This article provides a systematic review on liquid metal-enabled soft sensors as well as possible future development. Typical sensors made in this way include force sensors, temperature sensors, electrochemical sensors and so on. They work mainly depending on the alteration of resistance and capacitance of liquid metal. Electromagnetic metamaterials fabricated by liquid metal may propel the development of metamaterials and flexible metamaterial biosensors. With the excellent fluidity of liquid metal, those sensors show excellent stretchability, which have potential in wearable device and health-monitoring. Electrode arrays can be combined with soft sensors for large-area detection and sensitivity improvement. 

In summary, liquid metal has shown great potential in the area of soft sensors. Based on the progress achieved, more efforts are necessary and the efforts will be worthy. Future research may focus on resolution and repeatability as well as designing the testing principles of liquid metal-based sensors. Microsphere sensors based on liquid metal and metal-enabled liquid sensors will be important research fields in the future. It is believed that the soft sensors fabricated by liquid metal could be widely applied in diverse biomedical areas and soft robots.
